# Development and validation of a quantitative pcr array assay for the detection of viral sequences in breast cancer

**DOI:** 10.1016/j.jgeb.2026.100693

**Published:** 2026-04-16

**Authors:** Karina del Carmen Trujillo-Murillo, Angel Lugo-Trampe, Iram Pablo Rodríguez-Sánchez, Margarita L Martínez-Fierro, Concepción Cordero-Chaclan, Yaliana Tafurt-Cardona, Paúl Mendoza Pérez, Alejandra de Jesús Joo-Domínguez, Rodrigo De la Cruz-Calderón, Marisol Espinoza-Ruiz, Consuelo Chang-Rueda, Fanny Carmina Lee-Faviel

**Affiliations:** a Faculty of Human Medicine, Campus IV, Universidad Autónoma de Chiapas, Tapachula 30700, Mexico; b Molecular and Structural Physiology Laboratory, School of Biological Sciences, Universidad Autónoma de Nuevo León, San Nicolás de los Garza 66455, Mexico; c Molecular Medicine Laboratory, Unidad de Medicina Humana y Ciencias de la Salud, Universidad Autónoma de Zacatecas, Zacatecas 98160, Mexico; d High Specialty Regional Hospital “Ciudad Salud”, Tapachula 30700, Mexico; e Faculty of Chemistry Sciences, Campus IV, Universidad Autónoma de Chiapas, Tapachula 30700, Mexico

**Keywords:** Breast cancer, qPCR multiplex array, Viral detection, HPV, MMTV, EBV

## Abstract

**Objective:**

To develop and validate a quantitative PCR Multiplex Array assay for the detection of viral sequences in breast cancer (BC) tissue, and to assess its potential utility as a molecular research and diagnostic tool.

**Methods:**

A total of 172 formalin-fixed paraffin-embedded (FFPE) biopsy samples from patients with a histological diagnosis of BC, and 10 non-neoplastic breast tissue samples were collected at the High Specialty Regional Hospital “Ciudad Salud” (HRAECS). The study was approved by the Institutional Research and Ethics Committees. Samples were analyzed using a qPCR Multiplex Array assay. gBlocks Gene Fragments at 10 ng/µL were used as positive controls for each target virus.

**Results:**

Assay validation demonstrated high specificity, linearity (R^2^ > 0.98), amplification efficiencies within the 90–110% range, and reproducibility (CV < 5%), confirming the reliability of this method for viral detection in clinical tissue samples. Viral sequences were detected in 30% (51/172) of cases for HPV, 24% (41/172) for MMTV, and 0.6% (1/172) for EBV. Sanger sequencing confirmed 100% sequence identity with GenBank reference sequences in all positive samples.

**Conclusion:**

A qPCR Multiplex Array assay targeting 21 viral sequences was developed and validated, providing a reliable tool for the detection of cancer-associated viral sequences in BC tissue. Detection of viral sequences confirms their presence in tumor tissue but does not establish causality with carcinogenesis. Future studies integrating transcriptomic, functional, and clinical analyses are needed to clarify the role of these viruses in BC, including associations with clinicopathological features and potential therapeutic implications.

## Highlights

• A qPCR Multiplex Array assay for simultaneous detection of 21 viral sequences was developed.
• The assay demonstrated high specificity, linearity (R^2^ > 0.98), and reproducibility (CV < 10%).
• Amplification efficiencies within the 90–110% range confirmed reliable quantitative performance.
• HPV, MMTV, and EBV sequences were detected in 30%, 24%, and 0.6% of breast cancer cases.
• Sanger sequencing confirmed 100% sequence identity in all qPCR-positive samples.

## Graphical abstract

**Figure:**
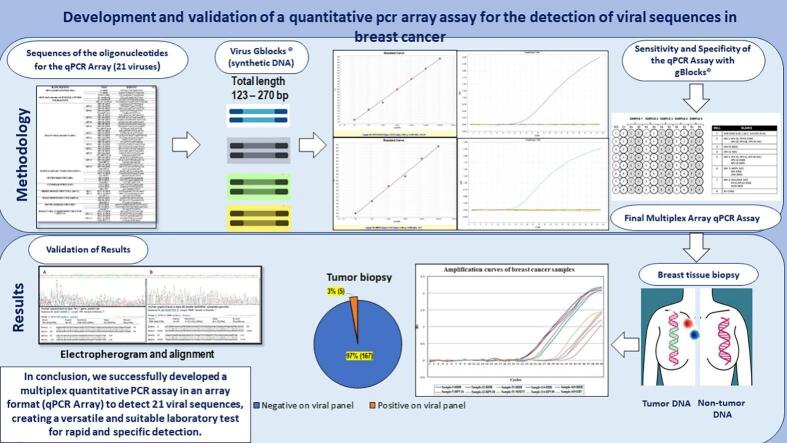

## 1 Introduction

According to the World Health Organization (WHO), approximately 2.3 million new cases of breast cancer (BC) were diagnosed worldwide in 2020, resulting in an estimated 685,000 deaths attributed to the disease.^1^ The International Agency for Research on Cancer (IARC) projects a 21% increase in new cases by 2030, corresponding to approximately 2.74 million affected women, with a global incidence rate of 47.8 cases per 100,000 women.^2^, ^3^ This increasing burden may be associated with a combination of environmental exposures and infectious agents, including viruses implicated in breast carcinogenesis.^4^ The IARC further highlights that a substantial proportion of human cancers have an infectious etiology. Oncogenesis may be driven by viral oncogenes, such as those of high-risk human papillomavirus (HPV), or by chronic inflammation induced by viruses such as hepatitis B (HBV) and hepatitis C (HCV), which together account for over 90% of infection-related cancers worldwide.^5^

The American Cancer Society estimates that the average lifetime risk of developing breast cancer for women in the United States is approximately 13%, meaning that 1 in 8 women will be diagnosed with the disease during their lifetime.^6^ In high-income countries, including the United States, Canada, and parts of Europe, most BC cases are detected and treated at early stages, contributing to mortality rate reductions of up to 30%,^7^ largely due to effective screening programs, early detection, and standardized molecular diagnostics. In contrast, in low- and middle-income countries such as Mexico, mortality rates remain high and continue to rise, primarily due to limited access to screening programs, delayed diagnosis, and a higher proportion of cases presenting at advanced stages.^8^

Molecular epidemiology studies have made it possible to track and estimate the contribution of infectious pathogens to the global cancer burden.^4^ Various molecular techniques, such as real-time quantitative polymerase chain reaction (qPCR), have proven helpful for early cancer detection, offering precision, cost-effectiveness, and high-throughput multiplex detection with greater specificity and sensitivity than conventional PCR methods.^9^ In this context, synthetic double-stranded DNA fragments (gBlocks) have been widely used as reliable standards and positive controls for qPCR assay validation, enabling accurate quantification across a broad dynamic range without the need for live viral material.^10^ Furthermore, multiplex molecular platforms have been previously applied for the simultaneous detection of multiple viral targets in oncological and infectious disease contexts, demonstrating significant advantages in throughput and resource efficiency compared to singleplex approaches, particularly in oncological and infectious disease diagnostics.^11^, ^12^

Multiple qPCR methods have been developed for the detection and quantification of viral genomes in cancer-related samples, providing valuable tools for the molecular diagnosis and monitoring of virus-associated malignancies.^11^, ^13^ To date, reports have focused on viral agents associated with the development of BC; however, these have generally been isolated, single-targeted studies.^13^ To the best of our knowledge, no comprehensive approach has been specifically designed to address the simultaneous detection of multiple viral sequences in this malignancy using a single real-time PCR reaction.

Therefore, it is essential to advance epidemiological research by investigating the mechanisms of viral transmission, establishment, and potential contribution to BC development, in order to determine whether these viral agents play a causal role in at least a proportion of BC cases. The objective of the present study was to develop and validate a quantitative PCR Multiplex Array assay for the simultaneous detection of viral sequences in breast cancer tissue, providing a robust tool for molecular diagnostics and research applications, while also exploring viral prevalence in a BC patient cohort from southern Mexico.

## 2 Materials and methods

### 2.1 Clinical samples

This study included a total of 172 formalin-fixed paraffin-embedded (FFPE) biopsies from patients with histologically confirmed breast cancer (BC) and 10 non-neoplastic breast tissue samples (fibroadenomas and normal breast tissue from reduction mammoplasties). Samples were stored at room temperature in the pathology archive of HRAECS until processing. The samples were obtained from patients treated at the HRAECS in Tapachula, Chiapas, Mexico. Clinical records and histopathological data were compiled into a database, including information obtained from patient questionnaires. All patient data were anonymized prior to analysis.

The study was reviewed and approved by HRAECS's Research and Ethics Committees (folio: 001/2011) and the Directorate General for Research and Postgraduate Studies (DGIP) of the Autonomous University of Chiapas (UNACH) in accordance with the Declaration of Helsinki. Participation was voluntary, and patient consent was documented through signed informed consent forms.

### 2.2 Total nucleic acid extraction

Total DNA was extracted from FFPE biopsy samples according to the manufacturer's instructions using the NucleoSpin DNA FFPE kit (Macherey-Nagel, Düren, Germany), which is specifically optimized for the recovery of fragmented DNA from formalin-fixed paraffin-embedded tissues. For RNA virus detection, total RNA was extracted from the same FFPE samples using the RNeasy FFPE Kit (Qiagen, Hilden, Germany), followed by reverse transcription using the High-Capacity cDNA Reverse Transcription Kit (Applied Biosystems, Thermo Fisher Scientific, Waltham, MA, USA) to generate complementary DNA (cDNA) for subsequent RT-PCR analysis.

Nucleic acid concentration and purity were assessed using a NanoDrop spectrophotometer (Thermo Fisher Scientific), with acceptable purity defined as A260/A280 ratios between 1.8 and 2.0 for DNA, and between 1.9 and 2.1 for RNA. DNA integrity was additionally evaluated by agarose gel electrophoresis. Only samples meeting these quality criteria were included in downstream analyses.

### 2.3 gBlocks and TaqMan probes

The target viruses for this assay were selected based on previously reported associations with human cancer, with particular emphasis on those implicated in breast carcinogenesis, including HPV, MMTV, EBV, CMV, HSV-1, HSV-2, HHV-8, BLV, HTLV-1, and HTLV-2. Selection criteria included published evidence of viral DNA or RNA detection in breast tumor tissue, proposed oncogenic mechanisms, and epidemiological relevance in the study population.

Viral sequences available in GenBank were analyzed using Beacon Designer 7.2, Primer Express 2.0, Oligo 7.0, and Amplify 3.1 to design oligonucleotides and TaqMan probes *in silico*, targeting conserved viral genome regions previously associated with human cancer. *In silico* specificity of each primer-probe set was validated using BLAST (https://blast.ncbi.nlm.nih.gov) against the GenBank nucleotide database to confirm unique target binding and to exclude potential cross-reactivity with human genomic sequences or other viral genomes included in the panel.

The use of fluorescently labeled probes (FAM, VIC, and NED) enabled the development of a Multiplex Quantitative PCR Array (qPCR Array) capable of simultaneously detecting 21 viral target sequences (Table 1). The assay also included a beta-globin (HBB) DNA positive control and an *E. coli* Aat gene internal reaction control to monitor DNA quality and reaction efficiency, respectively.

**Table 1:** Sequences of the oligonucleotides and TaqMan probes for the qPCR Array (Multiplex) assay design for the detection of 21 viruses.

Target Sequence		Name	Sequence	Fluorophore
Beta Globin (Control DNA)		C + -HBBF	CTCTGCCTATTGGTCTAT	
		C + -HBBR	GACTCTTGGGTTTCTGATAG	FAM
		FAM-C + -HBB	CCCAAAGGACTCAAAGAAC	
Gene Aat *Escherichia coli* (Internal Control For Reaction)		CI + -AatAEcoliF	TCTCACTAAGCATCTCAA	
		CI + -AatAEcoliR	ATCGGCTTATGAAGCAAAA	NED
		NED-CI + -AatAEcoli	TTCCGAGAACAAATTAATTATGTAG	
Human Papillomavirus (HPV)		HPV18F-QPCR	CAAGGAACATTTTGTGAAC	
	HPV18	HPV18R-QPCR	CGTGGACTTAACTCTGTA	
		HPV45F-QPCR	GACACACAATTATCCATTTG	FAM
	HPV45	HPV45R-QPCR	GCATCATTCTGAACTTCC	
		FAM-HPV18/45	CAGGCATTGTTCCA	
		HPV59F-QPCR	TGCAGGCAATAGTAGATAA	
	HPV59	HPV59R-QPCR	TGTGGTATCATCAATAAAGTC	
		HPV68F-QPCR	CAAGCAATAGTAGATAAACAAA	
	HPV68	HPV68R-QPCR	GTAGCATCATCAATGAAATC	VIC
		HPV39F-QPCR	CAGGCAATAGTAGATAAACAA	
	HPV39	HPV39R-QPCR	TGTGGAATCATCAATAAAGTC	
		VIC-HPV39/59/68	TCTGAACCTGTATCTGT	
		HPV35F-QPCR	GGTAGTGGTCTTTACATTTC	
	HPV35	HPV35R-QPCR	CCTGACACACACTTAAAC	
		HPV31F-QPCR	TCCAGTATATGAATTAAGTGATAA	NED
	HPV31	HPV31R-QPCR	TCCTGACACACATTTAAAC	
		NED-HPV35/31	TTGTCCTCTTCCTCGT	
		HPV52F-QPCR	CCCATTTCCATTTGATGA	
	HPV52	HPV52R-QPCR	CCTCTTCCTGTATTAAATCTAA	
		HPV33F-QPCR	GCTTCTTACCTCAAATACAA	
	HPV33	HPV33R-QPCR	GTCCTCTTCCTCTATTAAATC	VIC
		HPV58F-QPCR	GCACAGTAGACTAACAGTA	
	HPV58	HPV58R-QPCR	TGTCCTCTTCCTCTATTAAG	
		VIC-HPV33/52/58	TTGCACCACGTCCTT	
		HPV16F-QPCR	GTGTCTCCAATGTGTATG	
	HPV16	HPV16R-QPCR	GTACCATCTGTGATAATTCA	FAM
		FAM-HPV16	TACAGCAGCAGCAT	
		HPV56F-QPCR	AACGGAGGCTTATTTTATC	
	HPV56	HPV56R-QPCR	AGTCCTCACTATTGTTACTA	NED
		NED-HPV56	ACCTACAAGACAGC	
Murine Mammary Tumor Virus (MMTV)		MMTVF-QPCR	GAGTCAGCTCAAGAAAGC	
		MMTVR-QPCR	CGAGTGATGTGAAACTGAA	VIC
		VIC-MMTV	CGCACTACATCATCA	
Epstein-Barr Virus (EBV)		EBVF-QPCR	CTTCGAGGTGCATAGAAG	
		EBVR-QPCR	CGTTGTCAAACAGGACAA	FAM
		FAM-EBV	TTCCGTCAGTTCCA	
Cytomegalovirus (CMV)		CMVF-QPCR	CAACCGCTACTAGACAAG	
		CMVR-QPCR	CAGACACTCGTTGATTCG	NED
		NED-CMV	AACCAGGACTTGCT	
Herpes Simplex Virus Type 1 (HSV-1)	HSV-1	HSV1/2F-QPCR	CCTCRAAKATCCCCATAAAC	
	HSV-2	HSV2/1R-QPCR	GGTCTTCAAGGAGAACATC	VIC
		VIC-HSV1/2	AACCACACCTGCGA	
Human Herpesvirus Type 8 (HHV-8)		HHV8F-QPCR	GAGTGTGTAGTGCATCGA	
		HHV8R-QPCR	GCCGCACCTTAAAGATATG	NED
		NED-HHV8	TTCAACCTGGAGCA	
Bovine Leukemia Virus (BLV)		BLVF-QPCR	MNMYCYKDRSYKSYKSAYYTCACCT	
		BLVR-QPCR	TACCTGMCSSCTKSCGGATAGCCGA	FAM
		FAM-BLV	CTCAGCTCTCGGTCC	
Human T-Cell Lymphotropic Virus Type 1 (HTLV-1)		HTLV1F-QPCR	CACCAATTCCTCCACCAG	
	HTLV-1	HTLV1R-QPCR	GTCGCCTTGTACACAGTC	
		HTLV2F-QPCR	TCTCCTAACGGCAATCTC	FAM
	HTLV-2	HTLV2R-QPCR	ATCGGCCTGTACACAATC	
		FAM-HTLV1/2	CCAAACACGTAGAC	

gBlocks Gene Fragments (synthetic double-stranded DNA fragments) at a concentration of 10 ng/µL were used as positive controls for each target virus. All qPCR reactions were performed using TaqMan Fast Universal PCR Master Mix (2X) (Applied Biosystems, Thermo Fisher Scientific, Waltham, MA, USA). The TaqMan probe for the beta-globin (HBB) DNA positive control was labeled with FAM, while the probe for the *E. coli* Aat internal reaction control was labeled with NED (Table 1).

The analytical performance of each qPCR assay was subsequently evaluated for sensitivity, specificity, and reproducibility, with all experiments performed in triplicate under each condition.

### 2.4 Sensitivity

Standard curves were constructed to assess the linearity and efficiency of each qPCR assay using gBlocks at an initial concentration of 10 ng/µL. The sequence and length of each gBlock used are provided in Table 2. For each standard curve, eight 10-fold serial dilutions were prepared from each gBlock. Copy numbers were estimated based on the molecular weight of each template, assuming an average of 650 Da per base pair for double-stranded DNA. The calculated copy numbers for each dilution are provided in Supplementary Table 1. Standard curve parameters, including slope, y-intercept, coefficient of determination (R^2^), and amplification efficiency (E, calculated as E = [10^(−1/slope) − 1] × 100%), were determined by linear regression analysis of the Ct (cycle threshold) values obtained from each serial dilution. Acceptable amplification efficiency was defined as 90–110%, consistent with MIQE guidelines.

**Table 2:** GBlocks sequence and length.

Name	PB Length	Sequence OF G-Blocks	Number of Copies
HPV18-E1-REF	180	CACAAGGAACATTTTGTGAACAGGCAGAGCTAGAGACAGCACAGGCATTGTTCCATGCGCAGGAGGTCCACAATGATGCACAAGTGTTGCATGTTTTAAAACGAAAGTTTGCAGGAGGCAGCAAAGAAAACAGTCCATTAGGGGAGCGGCTGGAGGTGGATACAGAGTTAAGTCCACGGT	5.15E + 10
HPV45-E1-REF	143	GCAACAGATACAGGGTCGGATATGGTAGATTTTATTGACACACAATTATCCATTTGTGAACAGGCAGAGCAAGAGACAGCACAGGCATTGTTCCATGCGCAGGAAGTTCAGAATGATGCACAGGTGTTGCATCTTTTAAAACG	6.48E + 10
HPV59-E1-REF	135	GGATGGTTTTTTGTGCAGGCAATAGTAGATAAAAAAACAGGTGACAAAATTTCAGATGACGAGGATGAAAATGCAACAGATACAGGTTCAGACTTGGTAGACTTTATTGATGATACCACAACAATTTGTGTACAG	6.86E + 10
HPV68-E1-REF	135	GGATGGTTTTTTGTACAAGCAATAGTAGATAAACAAACAGGTGACACAGTCTCAGAGGATGAGGATGAAAACGCGACAGATACAGGTTCAGACATGGTAGATTTCATTGATGATGCTACAGATATTTGTATACAG	6.86E + 10
HPV39-E1-REF	135	GGATGGTTTCTAGTACAGGCAATAGTAGATAAACAAACAGGCGATACAGTGTCGGAGGATGAGGATGAAAATGCAACAGATACAGGTTCAGACTTGGCAGACTTTATTGATGATTCCACAGATATTTGTGTACAG	6.86E + 10
HPV35-E1-REF	187	AGGGTAGTGGTCTTTACATTTCACAATGAATTCCCATTTGATAAAAATGGAAACCCAGTGTATGGGCTTAATGATAAAAACTGGAAATCCTTTTTCTCAAGGACGTGGTGCAGATTAAATTTGCACGAGGAAGAGGACAAAGAAAATGATGGAGACGCTTTCCCAGCGTTTAAGTGTGTGTCAGGAC	4.95E + 10
HPV31-E1-REF	160	CGGAAATCCAGTATATGAATTAAGTGATAAAAACTGGAAATCCTTTTTCTCAAGGACGTGGTGCAGATTAAATTTGCACGAGGAAGAGGACAAAGAAAACGATGGAGACTCTTTCTCAACGTTTAAATGTGTGTCAGGACAAAATATTAGAACATTATGA	5.79E + 10
HPV52-E1-REF	153	GGTCGTGTTTCATTTCAAAAACCCATTTCCATTTGATGAAAATGGCAATCCCATATATGAAATTAACAACGAAAATTGGAAATCCTTTTTCTCAAGGACGTGGTGCAAATTAGATTTAATACAGGAAGAGGACAAGGAAAACGATGGAGTCGA	6.06E + 10
HPV33-E1-REF	200	CTGCTTCTTACCTCAAATACAAATGCAGGCACAGACTCTAGATGGCCATATTTACATAGTAGATTAACAGTATTTGAATTTAAAAATCCATTCCCATTTGATGAAAATGGTAACCCAGTGTATGCAATAAATGATGAAAATTGGAAATCCTTTTTCTCAAGGACGTGGTGCAAATTAGATTTAATAGAGGAAGAGGACAA	4.63E + 10
HPV58-E1-REF	150	TTGCACAGTAGACTAACAGTATTTGAATTTAACAATCCATTTCCATTTGATGCAAATGGTAATCCAGTGTATAAAATAAATGATGAAAATTGGAAATCCTTTTTCTCAAGGACGTGGTGCAAATTAGGCTTAATAGAGGAAGAGGACAAG	6.18E + 10
HPV16-E1-REF	214	GCTGTCTAAACTATTATGTGTGTCTCCAATGTGTATGATGATAGAGCCTCCAAAATTGCGTAGTACAGCAGCAGCATTATATTGGTATAAAACAGGTATATCAAATATTAGTGAAGTGTATGGAGACACGCCAGAATGGATACAAAGACAAACAGTATTACAACATAGTTTTAATGATTGTACATTTGAATTATCACAGATGGTACAATGGGCC	4.33E + 10
HPV56-E1-REF	169	AAAACGGAGGCTTATTTTATCAGACCTACAAGACAGCGGGTATGGCAATACATTGGAAACTCTGGAAACACCAGAACAGGTAGATGAAGAGGTACAGGGACGTGGGTGCGGGAATACACAAAATGGAGGCTCACAAAACAGTACCTATAGTAACAATAGTGAGGACTCT	5.48E + 10
EBV-BNRF1-REF	150	CTGGGCGCCACGTACGTGCTTCGCGTGGAGATGGGCAGGCAGTTTGGCTTCGAGGTGCATAGAAGCCGGCCCTCCTTCCGTCAGTTCCAGGCCATCAATCACCTTGTCCTGTTTGACAACGCCCTTCGCAAGTACGATTCCGGCCAGGTG	6.18E + 10
MMTV-REF	197	GGCACAGGGAAATGCCTATGCAGATTCTTTAACAAGAATTCTGACCGCTTTAGAGTCAGCTCAAGAAAGCCACGCACTACATCATCAAAATGCCGCGGCGCTTAGGTTTCAGTTTCACATCACTCGTGAACAAGCGCGAGAAATAGTAAAATTATGTCCCAATTGCCCCGACTGGGGGCACGCGCCGCAATTAGGGG	4.70E + 10
CMV-UL48-REF	200	GCACCAACCACTGGAGGCCACACTCGAGCTGCTCTTGGGCCTAGATCAACGCGCCCAACCGCTACTAGACAAGTTCAACCAGGACTTGCTGTCGGCGCTGCAGCAGCTGAGCAAAAAACTAGACGGGCGAATCAACGAGTGTCTGCACGGCGTGCTGACGGGTGATGTAGAGCGGCGCTGTCACCCGCACCGAGAAGCGG	4.63E + 10
HTLV1-TAX-REF	270	CCTCCTCCTCCTTGTCCTTTAACTCTTCCTCCAAGGATAATAGCCCGTCCACCAATTCCTCCACCAGCAGGTCCTCCGGGCATGGAACAGGCAAACATCGAAACAGCCCTACGGATACAAAGTTAACCATGCTTATTATCAGCCCACTTCCCAGGGTTTGGACAGAGTCTTCTTTTCGGATACCCAGTCTACGTGTTTGGAGACTGTGTACAAGGCGACTGGTGCCCCATCTCTGGGGGACTATGTTCGGCCCGCCTACATCGTCACGCC	3.43E + 10
HTLV2-TAX-REF	270	AGGGCGACCTTTTGCTGTCCTTCTCGGTTCCTCTCCAGGGGGAGGCACACCAGATGTCAGACTCGCCTCTCCCTGGTCTCCTAACGGCAATCTCCTAAAATAGTCTAAAAAATCACACATAATTACAATCCTGTCTCCTCTCAGCCCATTTCCCAGGATTTGGACAGAGCCTCCTATATGGATACCCCGTCTACGTGTTTGGCGATTGTGTACAGGCCGATTGGTGTCCCGTCTCAGGTGGTCTATGTTCCACCCGCCTACATCGACATG	3.43E + 10
HHV8-UL27-REF	270	GCCCCCACCAAACCTGGTGAGGAAGCATCTGGTCCTAAGAGTGTGGACTTTTACCAGTTCAGAGTGTGTAGTGCATCGATCACCGGGGAGCTTTTTCGGTTCAACCTGGAGCAGACGTGCCCAGACACCAAAGACAAGTACCACCAAGAAGGAATTTTACTGGTGTACAAAAAAAACATAGTGCCTCATATCTTTAAGGTGCGGCGCTATAGGAAAATTGCCACCTCTGTCACGGTCTACAGGGGCTTGACAGAGTCCGCCATCACCAAC	3.43E + 10
HSV1-UL27-REF	250	GCGCACGTACTTGGCCGTGGACCGACAGACCCCCTTGGCGTTGATCTTGTCGATCACCTCCTCGAAGGGGACGGGGGCGCGGTCCTCAAAGATCCCCATAAACTGGGAGTAGCGGTGGCCGAACCACACCTGCGAAACGGTGACGTCTTTGTAGTACATGGTGGCCTTGAACTTGTACGGGGCGATGTTCTCCTTGAAGACCACCGCGATGCCCTCCGTGTAGTTCTGACCCTCGGGCCGGGTCGGGCAG	3.71E + 10
HSV2-UL27-REF	123	GTCCTCGAATATCCCCATAAACTGGGAGTAGCGGTGGCCGAACCACACCTGCGACACGGTCACGTCTTTGTAGTACATGGTGGCCTTGAATTTGTACGGGGCGATGTTCTCCTTGAAGACCAC	7.53E + 10
CI + -AatAEcoli	126	CTTCTATCGGCTTATGAAGCAAAAATGCAAAATGATAAATTGGATATTGCTAATTATTTAAAATATATTGAGATGCTTAGTGAGAGGAATAGCTACATAATTAATTTGTTCTCGGAAATCATTAAC	7.35E + 10
CTRL-HHB	270	TGGGCAGGTTGGTATCAAGGTTACAAGACAGGTTTAAGGAGACCAATAGAAACTGGGCATGTGGAGACAGAGAAGACTCTTGGGTTTCTGATAGGCACTGACTCTCTCTGCCTATTGGTCTATTTTCCCACCCTTAGGCTGCTGGTGGTCTACCCTTGGACCCAGAGGTTCTTTGAGTCCTTTGGGGATCTGTCCACTCCTGATGCTGTTATGGGCAACCCTAAGGTGAAGGCTCATGGCAAGAAAGTGCTCGGTGCCTTTAGTGATGGC	3.43E + 10

### 2.5 Specificity

The specificity of each qPCR assay was evaluated by cross-reactivity testing, in which each primer and probe mix (at an initial concentration of 20×) was challenged against its corresponding gBlock and all other gBlocks included in the panel (at a 1:1000 dilution). For example, the FAM-EBV qPCR assay was tested against its corresponding EBV-REF gBlock as well as against all other gBlocks in the panel to assess cross-reactivity (Table 2). This approach was applied to each qPCR assay in the array. The limit of detection (LOD) for each assay was determined as the lowest copy number concentration that yielded a detectable and reproducible signal in all triplicate reactions.

### 2.6 Reproducibility

Intra- and inter-assay reproducibility were evaluated using selected serial dilutions of each gBlock. Intra-assay reproducibility was assessed by analyzing each dilution in triplicate within the same run, while inter-assay reproducibility was determined by repeating the analysis on two independent days. The coefficient of variation (CV) was calculated as the ratio of the standard deviation to the mean of the Ct values, both within the same run (intra-assay) and across independent runs on different days (inter-assay), as described in Supplementary Table 1.

Following the individual analytical validation of each assay, multiplex combinations of qPCR assays were tested to optimize simultaneous detection performance within the array format. Optimization of multiplex reactions included evaluation of primer and probe concentrations, assessment of amplification efficiency and competitive inhibition between oligonucleotides, and verification of the absence of non-specific amplification and primer-dimer formation. Singleplex and multiplex performance parameters were compared to confirm that multiplexing did not significantly compromise assay sensitivity or specificity. All amplification reactions were performed using a 7500 Fast Real-Time PCR System (Applied Biosystems, Thermo Fisher Scientific, Waltham, MA, USA).

### 2.7 Detection of viruses by quantitative PCR

#### 2.7.1 Controls used for virus detection

A beta-globin (HBB) gBlock (270 bp) was used as a DNA quality control, and an *E. coli* Aat gene gBlock® (126 bp) served as an internal reaction control, as previously described (Section 2.3). Virus-specific gBlocks (Table 2) were diluted 1:1000 (1 µL in 999 µL of nuclease-free H_2_O) from a stock concentration of 10 ng/µL prior to use as positive controls (Supplementary Table 1). Detailed amplification reaction conditions and thermocycler program settings are provided in Supplementary Tables 2 and 3, respectively.

The qPCR Multiplex Array was designed to detect viral DNA sequences in separate multiplexed reactions per fluorophore channel (FAM, VIC, NED), with each reaction containing up to three viral targets simultaneously detected through their respective labeled probes. This array configuration allows the analysis of five clinical samples in triplicate per run, including all controls.

#### 2.7.2 Detection of viral sequences by qPCR multiplex array

Clinical breast tissue samples were analyzed individually using the qPCR Multiplex Array assay under standard amplification conditions. Prior to analysis, all extracted DNA samples were normalized to a concentration of 100 ng/µL to ensure consistency across reactions.

Each 20 µL reaction consisted of 10 µL of TaqMan Fast Universal PCR Master Mix (2×) (Applied Biosystems, Thermo Fisher Scientific, Waltham, MA, USA), 1 µL of the corresponding primer and TaqMan probe mix (20×, containing each primer at a final concentration of 900 nM and each probe at 250 nM, following standard TaqMan assay recommendations), 1 µL of normalized DNA template (100 ng/µL), and 8 µL of nuclease-free water. Each reaction also included the beta-globin (HBB) DNA positive control and the *E. coli* Aat gene internal reaction control, as described in Section 2.7.1.

Amplification was performed on a 7500 Fast Real-Time PCR System (Applied Biosystems, Thermo Fisher Scientific, Waltham, MA, USA) using the standard fast cycling conditions recommended by the manufacturer: an initial denaturation step at 95°C for 20 s, followed by 40 cycles of 95°C for 3 s (denaturation) and 60°C for 30 s (annealing/extension). Fluorescence data were collected at the annealing/extension step of each cycle.

A sample was considered positive for a given viral target when a Ct value was detected within the linear range of the corresponding standard curve, in the presence of a valid internal reaction control signal. Specifically, a Ct value <38 in all triplicates was classified as positive, while samples yielding no amplification signal (undetermined Ct) or Ct ≥38 were classified as negative. All clinical samples were run in duplicate to ensure result reliability. Following assay validation, the complete panel of 172 BCE samples and 10 non-neoplastic control samples was analyzed using the final optimized multiplex format.

### 2.8 Sanger sequencing for results validation

To confirm the identity of qPCR-positive amplicons, Sanger sequencing was performed on all positive samples. Positive amplicons were sequenced directly and bi-directionally in triplicate using M13 universal primers (M13GP5+/M13GP6+) and the BigDye Terminator v3.1 Cycle Sequencing Kit (Applied Biosystems, Thermo Fisher Scientific, Waltham, MA, USA), followed by capillary electrophoresis on an ABI 310 Genetic Analyzer (Applied Biosystems, Thermo Fisher Scientific, Waltham, MA, USA), according to the manufacturer's instructions. The sequences obtained were aligned to generate a consensus sequence and compared against the GenBank database using the BLAST algorithm (https://blast.ncbi.nlm.nih.gov).

### 2.9 Statistical analysis

Data were summarized using descriptive statistics, including percentages, means, and standard deviations. Coefficients of variation (CV) were calculated to assess intra- and inter-assay reproducibility, using the formula CV(%) = (SD/mean) × 100. Differences in viral prevalence between groups were assessed using the chi-square test or Fisher's exact test, as appropriate. A p-value < 0.05 was considered statistically significant. All statistical analyses were performed using IBM SPSS Statistics, version 25 (IBM Corp., Armonk, NY, USA).

## 3 Results

### 3.1 Demographic and clinical characteristics

Table 3 presents the demographic and clinical characteristics of the 172 patients with BC included in the study. The mean age of the population was 50 ± 11 years, with a mean weight of 67 ± 14 kg and a mean BMI of 29 ± 6 kg/m^2^. Approximately 10% of the women reported a family history of breast cancer and 10% reported a family history of cervical cancer, while an additional 22% reported other types of cancer among first-degree relatives. Twenty-seven percent of the patients were premenopausal at the time of diagnosis, and 22% were nulliparous.

**Table 3:** Demographic and clinical characteristics of patients with BC.

Characteristics	Women with BC (n = 172)
Demographics	
Age (years)	50 ± 11
Weight (kg)	67 ± 14
BMI (kg/m^2^)	29 ± 6
Family history	
Breast cancer	18 (10.46%)
Cervical cancer	18 (10.46%)
Other types of cancer	36 (21.93%)
Gynecological and obstetric history	
Premenopausal	46 (26.74%)
Postmenopausal	30 (17.44%)
Nulliparity	36 (21.93%)
Educational level	
Illiterate	26 (15.11%)
Primary school	86 (50.00%)
Secondary school	28 (16.27%)
High school	18 (10.46%)
Bachelor's degree	14 (8.14%)
Histological subtype of BC	
*In situ* carcinoma	4 (2.32%)
Ductal carcinoma	2 (50.00%)*
Lobulillar carcinoma	2 (50.00%)*
Invasive Carcinoma	168 (97.67%)
Invasive ductal carcinoma	94 (55.95%)**
Invasive lobular carcinoma	2 (1.19%)**
Infiltrating canalicular carcinoma	60 (35.71%)**
Infiltrating tubular/cribriform carcinoma	4 (2.38%)**
Infiltrating colloid carcinoma	6 (3.57%)**
Infiltrating papillary carcinoma	2 (1.19%)**

*Percentage calculated within the in situ carcinoma subgroup (n = 4).

**Percentage calculated within the invasive carcinoma subgroup (n = 168).

Regarding educational level, the majority of patients had a low level of formal education, with 15% being illiterate and 50% having completed only primary school. With respect to the histological subtype of BC, invasive carcinoma was the predominant diagnosis, accounting for 97.67% of cases (168/172). Among invasive carcinomas, invasive ductal carcinoma was the most frequent subtype (55.95%), followed by infiltrating canalicular carcinoma (35.71%), infiltrating colloid carcinoma (3.57%), infiltrating tubular/cribriform carcinoma (2.38%), and invasive lobular carcinoma and infiltrating papillary carcinoma (1.19% each). In situ carcinoma was identified in 2.32% of cases (4/172), comprising equal proportions of ductal and lobular subtypes.

It is important to note that data on additional clinicopathological parameters, including hormonal receptor status (ER, PR, HER2), tumor size, lymph node involvement, clinical stage, and histological grade, were not systematically available for all patients in this cohort. The absence of these variables represents a limitation of the current study, and future work will incorporate these parameters to assess potential associations between viral detection and disease characteristics.

### 3.2 Sensitivity, specificity, and limit of detection of the qPCR assay with gBlocks

**Fig. 1:**
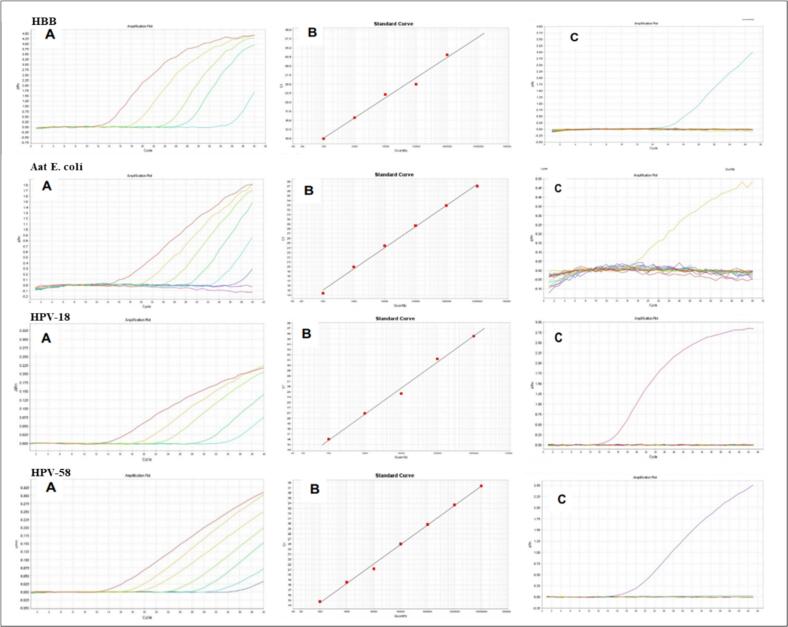
Sensitivity and specificity of the qPCR Assay for HBB, *E. coli* Aat, HPV-18, and HPV-58. A) Amplification curves of the gBlocks serial dilutions, showing well-separated Ct values corresponding to each dilution. B) Standard curves obtained by plotting Ct values against the log of the copy number for each dilution; R^2^ values indicate high linearity for all assays. C) Amplification curves obtained from each qPCR assay tested against its corresponding gBlock (positive signal) and against non-target gBlocks (no amplification observed), confirming assay specificity. Controls: HBB (human DNA control) and *E. coli* Aat (internal reaction control).

Fig. 1 shows the sensitivity and specificity results of the qPCR assay for the HBB control, *E. coli* Aat internal control, and HPV types 18 and 58. Standard curves were constructed and linearity was determined for the HBB control, *E. coli* Aat internal control, and all 21 viral targets included in the array (Supplementary Fig. 2).

The amplification curves (Fig. 1A) demonstrate well-separated Ct (cycle threshold) values across all serial dilutions, consistent with proportional decreases in template copy number.

The standard curves (Fig. 1B) showed high linearity, with R^2^ values of 0.985 for HBB, 0.997 for *E. coli* Aat, 0.983 for HPV-18, and 0.997 for HPV-58, all exceeding the accepted threshold of R^2^ > 0.98 for reliable qPCR assay performance.^10^ Amplification efficiencies were 97.2% for HBB, 99.1% for *E. coli* Aat, 95.4% for HPV-18, and 98.7% for HPV-58, all within the acceptable range of 90–110%. The limit of detection (LOD) was determined to be 10^3^ copies/µL for all assays included in the array, corresponding to the lowest serial dilution at which consistent and reproducible amplification was observed across all triplicate reactions.

Cross-reactivity testing (Fig. 1C) demonstrated that no non-specific amplification was observed when each primer-probe mix (20×) was challenged against non-target gBlocks (1:1000 dilution), confirming the specificity of each qPCR assay in the array.

### 3.3 Reproducibility of the qPCR assay with gBlocks

Four serial dilutions of gBlocks were selected to assess intra- and inter-assay reproducibility, corresponding to copy number concentrations of 10^3^, 10^4^, 10^5^, and 10⁶ copies/µL. All assays were performed in triplicate for each dilution in every run.

Table 4 presents the intra- and inter-assay coefficients of variation (CV) calculated from the Ct values for the HBB control, *E. coli* Aat internal control, and HPV types 18 and 58. The intra-assay CVs ranged from 0.69% to 6.22%, while the inter-assay CVs ranged from 0.79% to 6.12%. These results demonstrate good intra- and inter-assay reproducibility, as all CV values remained below the accepted threshold of 10% for routine molecular diagnostic testing.^23^, ^24^

**Table 4:** Intra- and inter-assay reproducibility of the qPCR assay for HBB, *E. coli* Aat, HPV-18, and HPV-58.

Sample	Dilution	Mean ± SD (intra-assay)	CV% (intra-assay)	Mean ± SD (inter-assay)	CV% (inter-assay)
HBB	10^3^	15.37 ± 0.44	2.84	15.00 ± 0.33	2.17
	10^4^	22.00 ± 1.06	4.82	22.41 ± 1.03	4.6
	10^5^	24.96 ± 0.17	0.69	25.14 ± 0.20	0.79
	10^6^	32.66 ± 0.60	1.83	31.95 ± 0.71	2.22
Aat E. coli	10^3^	14.62 ± 0.78	5.34	14.73 ± 0.67	4.52
	10^4^	20.55 ± 0.91	4.42	20.83 ± 0.76	3.67
	10^5^	24.34 ± 0.41	1.67	24.57 ± 0.45	1.84
	10^6^	28.55 ± 0.51	1.78	28.47 ± 0.55	1.93
HPV-18	10^3^	16.07 ± 0.63	3.93	16.18 ± 0.48	2.97
	10^4^	21.25 ± 0.64	3.00	22.08 ± 0.83	3.76
	10^5^	24.85 ± 0.25	0.99	25.40 ± 0.48	1.9
	10^6^	31.36 ± 0.56	1.78	31.89 ± 0.71	2.24
HPV-58	10^3^	15.65 ± 0.97	6.22	16.56 ± 0.85	5.10
	10^4^	19.21 ± 1.13	5.88	20.37 ± 1.25	6.12
	10^5^	23.61 ± 1.20	5.09	24.49 ± 0.77	3.12
	10^6^	26.15 ± 0.36	1.38	26.93 ± 0.75	2.79

CVa: intra-assay coefficient of variation (%); CVb: inter-assay coefficient of variation (%). SD: standard deviation. Ct: cycle threshold.

### 3.4 Application of the Multiplex qPCR assay

**Fig. 2:**
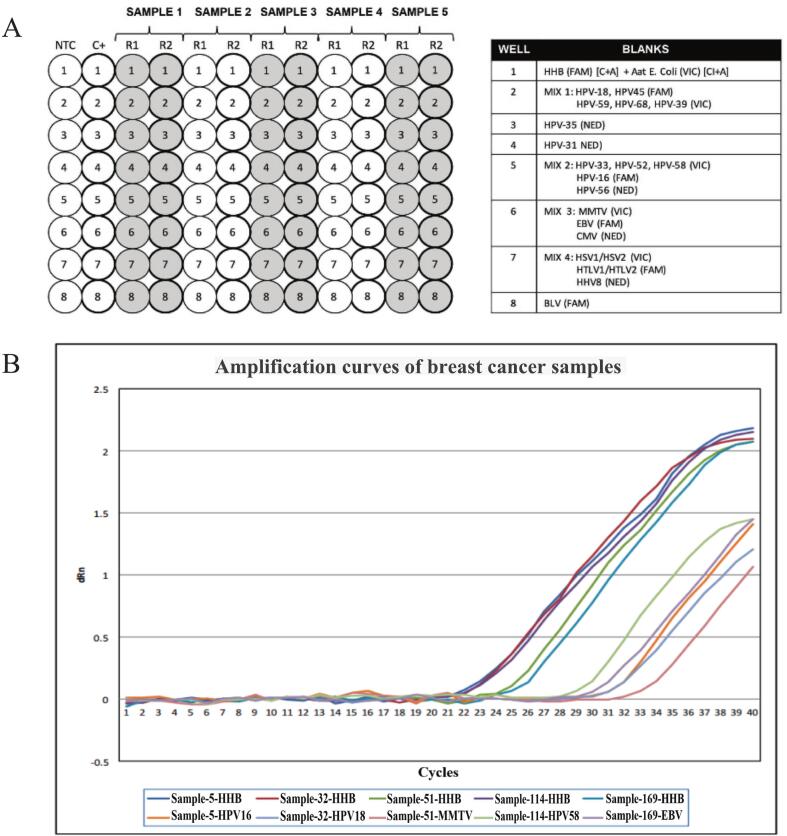
Application of the qPCR multiplex array assay to breast cancer samples. A) Final qPCR Array Multiplex configuration. The array layout allows for the simultaneous analysis of five samples in triplicate, including a human DNA positive control (HBB) and an internal amplification control (*E. coli* Aat). A sample is considered positive when the Ct (cycle threshold) value is <38 in all triplicates, while a Ct value ≥38 is classified as negative. NTC: No Template Control. [C+]: Human DNA Control (HBB). [CI+]: Internal Amplification Control (*E. coli* Aat). B) Representative amplification curves showing the detection of viral sequences in five breast cancer samples using the qPCR Multiplex Array assay. Amplification of the HBB DNA control was confirmed in all analyzed samples.

The final qPCR Multiplex Array design was optimized by testing multiple assay combinations to evaluate amplification efficiency, potential oligonucleotide competition, non-specific amplification, primer-dimer formation, and overall detection performance. This process resulted in a versatile and robust array configuration capable of simultaneously detecting 21 viral target sequences in a single reaction (Fig. 2A).

The optimized qPCR Multiplex Array assay was initially applied to a representative subset of five breast cancer samples to demonstrate assay performance under clinical conditions. Valid HBB DNA control signals were confirmed in all five reactions, ensuring DNA quality and reaction integrity. Viral sequences were successfully detected in each of the five samples: HPV-16 (patient #5), HPV-31 (patient #32), MMTV (patient #51), HPV-58 (patient #114), and EBV (patient #169) (Fig. 2B).

Following validation of the array performance in the initial subset, the assay was applied to the complete set of 172 breast cancer samples and 10 non-neoplastic control samples using the final optimized multiplex format. Viral sequences were detected in 30% of BC cases for HPV (51/172), 24% for MMTV (41/172), and 0.6% for EBV (1/172). No viral sequences were detected in the non-neoplastic control samples.

### 3.5 Validation of results by Sanger sequencing

Sanger sequencing was performed on all qPCR-positive samples to confirm the identity of the detected viral sequences, as described in Section 2.8. The sequences obtained from each sample were aligned and curated to generate a consensus sequence, which was subsequently compared against the GenBank database using the BLAST algorithm (https://blast.ncbi.nlm.nih.gov). Sequence identity was confirmed in 100% of the qPCR-positive samples analyzed, corresponding to 93 unique amplicons sequenced in triplicate: 51 from HPV, 41 from MMTV, and 1 from EBV.

**Fig. 3:**
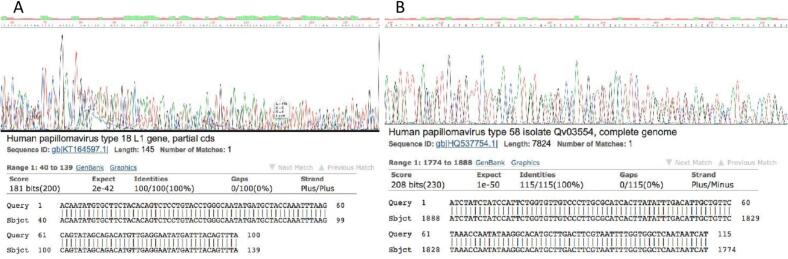
Sanger sequencing validation of qPCR-positive samples. Representative electropherograms and BLAST alignment results for two breast cancer samples positive for HPV-18 (A) and HPV-58 (B). Both samples show 100% sequence identity with their respective GenBank reference sequences, confirming the specificity of the qPCR Multiplex Array assay for viral sequence detection.

Among the HPV-positive cases, the most frequently identified genotypes were HPV-16 and HPV-18, followed by HPV-58. The sequence identity of all qPCR-positive samples for MMTV and EBV was likewise confirmed at 100%. Fig. 3 shows two representative electropherograms and their corresponding BLAST alignment results for HPV-18 and HPV-58, illustrating the high sequence identity obtained with GenBank reference sequences.

## 4 Discussion

The qPCR Multiplex Array assay developed in this study enables the simultaneous detection of 21 viral target sequences in breast cancer (BC) biopsy samples, representing, to the best of our knowledge, one of the first comprehensive platforms specifically designed for this purpose in BC tissue. Several studies have reported that infectious agents, including HPV, MMTV, EBV, HBV, HCV, and HTLV-1, are associated with the development of specific cancers, including BC.^10^, ^11^ However, the role of these viruses in tumorigenesis has not been fully established; they appear to be involved in certain key steps of cancer development rather than acting as sole causative agents.^13^

An important consideration when interpreting these results is that the detection of viral DNA sequences in tumor tissue does not necessarily indicate active infection or a causal role in carcinogenesis. The presence of viral sequences may reflect latent infection, viral persistence, integration into the host genome, or the incidental detection of virus-harboring infiltrating immune cells rather than direct infection of tumor cells.^14^ To confirm true viral infection and establish its biological relevance in BC, additional evidence is required, such as viral gene expression at the mRNA or protein level, demonstration of viral genome integration into host chromosomal DNA, or evidence of active viral replication. Therefore, the viral prevalence data reported in this study should be interpreted as evidence of viral DNA presence, not as proof of etiological involvement.

In our study, viral sequences were detected in 30% (51/172) of cases for HPV, 24% (41/172) for MMTV, and 0.6% (1/172) for EBV. Comparable detection rates for HPV and MMTV were reported by Naushad et al. in Pakistan, where HPV was detected in 18% (45/250) and MMTV in 29% (83/250) of BC cases; however, EBV prevalence in that study (24%, 60/250) was notably higher than the 0.6% observed in the present study.^15^ Similar findings have been reported in other geographic regions; Tadlaoui et al. detected HPV in 45.7%, MMTV in 72.9%, and EBV in 67.1% of invasive breast cancer cases in Morocco, further highlighting the geographic variability in viral prevalence across populations.^16^ The markedly lower EBV prevalence in our cohort compared to some international studies warrants commentary. This discrepancy may be attributable to several factors, including geographic and ethnic differences in EBV strain distribution, variation in detection methodology and assay sensitivity, differences in tissue type and sample preparation (particularly FFPE-associated DNA degradation), and population-specific differences in EBV latency patterns. The lower prevalence may also reflect genuine biological differences in EBV involvement in BC among Mexican women from southern Mexico. Standardized, multicenter studies using uniform detection platforms will be essential to clarify EBV's epidemiological role in BC across diverse populations.

Although the complete pathogenic and oncogenic roles of these viruses in BC development remain to be fully elucidated, it has been proposed that HPV and MMTV may integrate into the host cell genome, potentially activating oncogenes and inactivating tumor suppressor genes.^15^, ^17^ The biological significance of the HPV prevalence rates observed in this study is further supported by a recent systematic review and meta-analysis reporting that HPV DNA is significantly more prevalent in BC tissue than in normal breast controls (OR = 3.83; 95% CI 2.03–7.25).^18^ These mechanisms may contribute to the initiation and progression of BC in a subset of cases. In Mexico, previously reported detection rates in BC patients vary by study: HPV at 10% (6/60),^19^ MMTV at 12% (57/458),^20^ and EBV at 7% (4/86).^21^ The prevalence rates observed in the present study are higher for HPV and MMTV compared to these prior Mexican reports, which may reflect differences in geographic distribution, sample size, or detection methodology. Notably, none of these viruses were detected in the non-neoplastic control samples included in the present study, providing important evidence that the detected signals are not attributable to non-specific amplification or sample contamination.

The biological significance of the HPV and MMTV prevalence rates observed in this study should be contextualized within the existing literature. For HPV, the predominance of HPV-16 and HPV-18 genotypes in our cohort is consistent with their known oncogenic potential and their documented detection in BC tissue in multiple geographic regions.^20^ For MMTV, the detection of MMTV-like sequences in approximately one quarter of BC cases aligns with reports from several countries and supports the hypothesis that MMTV-related retroviruses may play a role in a subset of human BC cases, though a causal relationship remains to be established.^15^, ^20^

Numerous molecular techniques have been developed for the identification of oncogenic viruses in clinical samples.^22^, ^23^ Unlike conventional PCR, which provides qualitative, single-target detection, multiplex qPCR enables the simultaneous quantification of multiple DNA targets in a single reaction, offering significant advantages in terms of throughput, sensitivity, and resource efficiency.^24^ The assay developed in this study represents a simple, sensitive, and cost-effective tool amenable to implementation in standard molecular diagnostic laboratories. The array format enables the detection of 21 cancer-associated viral target sequences through optimized multiplex qPCR combinations in a single, highly versatile reaction configuration (Table 1).

The specificity, sensitivity, and reproducibility assessments conducted using gBlocks demonstrated that this method is reliable for the detection of cancer-associated viral sequences in clinical tissue samples. The analytical evaluation confirmed the absence of competitive inhibition among oligonucleotides and TaqMan probes, with no non-specific amplification or primer-dimer formation observed. These findings are consistent with those reported by Li et al. and Laamiri et al., who demonstrated the reliability of multiplex RT-qPCR assays for the simultaneous detection of co-infecting avian viruses.^25^, ^26^, ^27^ Regarding sensitivity and linearity, the standard curves for all assays demonstrated high R^2^ values exceeding 0.98 and amplification efficiencies within the 90–110% range, consistent with reliable quantitative performance across the tested copy number range.^28^, ^29^ The intra-assay CVs ranged from 0.69% to 6.22%, and the inter-assay CVs ranged from 0.79% to 6.12%, all remaining well below the accepted threshold of 10%. Sanger sequencing confirmed 100% sequence identity in all qPCR-positive samples (93 unique amplicons: 51 HPV, 41 MMTV, and 1 EBV), validating the specificity of the multiplex qPCR array assay.

Regarding clinicopathological parameters, the current study focused primarily on assay development and initial clinical application. The incorporation of variables such as hormonal receptor status (ER, PR, HER2), tumor subtype, clinical stage, histological grade, tumor size, and lymph node involvement would be valuable in future studies to explore potential associations between viral detection and disease characteristics. This represents an important avenue for further research that the authors intend to pursue in a dedicated follow-up study.

Despite the strengths of this study, several limitations should be acknowledged. First, the cross-sectional study design does not allow for causal inference between viral presence and BC development. Second, the use of FFPE tissue may result in fragmented or chemically modified nucleic acids that could affect detection sensitivity, a factor that should be considered when comparing results with studies using fresh-frozen tissue. Third, the lack of functional validation (e.g., viral gene expression, protein detection, integration analysis) limits the biological interpretation of the detected viral sequences. Fourth, the absence of complete clinicopathological data restricts the ability to assess clinical correlations. Finally, technical limitations inherent to PCR-based detection, such as susceptibility to contamination or detection of non-replicative viral DNA from infiltrating immune cells, should also be considered.

Overall, this study provides a robust methodological framework for the simultaneous detection of multiple viral sequences in BC tissue, while emphasizing the need for cautious interpretation and further investigation into the biological and clinical implications of these findings.

## 5 Conclusion

A qPCR Multiplex Array assay targeting 21 viral sequences was developed and validated for the simultaneous detection of cancer-associated viral sequences in breast cancer (BC) tissue. The assay demonstrated high specificity, linearity (R^2^ > 0.98), amplification efficiencies within the 90–110% range, and reproducibility (CV < 10%), confirming its reliability as a sensitive, versatile, and cost-effective tool for molecular research and potential diagnostic applications in oncology.

While the detection of viral DNA confirms the presence of viral sequences in BC tumor tissue, it does not establish a causal relationship with carcinogenesis. These findings should therefore be interpreted as evidence of methodological advancement and viral prevalence documentation rather than etiological confirmation.

Future research should integrate molecular, functional, and clinical approaches to better understand the role of viruses in BC development. Specifically, the following directions are proposed: (1) evaluation of viral gene expression at the mRNA and protein levels to distinguish active from latent infection; (2) assessment of viral genome integration into host chromosomal DNA using next-generation sequencing approaches; (3) correlation of viral detection with clinicopathological features such as hormonal receptor status, tumor subtype, histological grade, and clinical stage; (4) large-scale multicenter studies in diverse geographic and ethnic populations to validate the prevalence findings and establish population-specific risk profiles; and (5) exploration of potential antiviral or targeted therapeutic strategies, should a causal role for specific viruses be established in a subset of BC cases.

The implementation of standardized, sensitive, and cost-effective detection platforms, such as the qPCR Multiplex Array assay developed in this study, may contribute to advancing research on virus-associated cancers and improving molecular diagnostic strategies, particularly in low- and middle-income settings where access to comprehensive diagnostic tools remains limited.

## CRediT authorship contribution statement

**Karina del Carmen Trujillo-Murillo:** Writing – review & editing, Methodology, Investigation. **Angel Lugo-Trampe:** Writing – original draft, Formal analysis, Conceptualization, Methodology, Software, Writing – review & editing. **Iram Pablo Rodríguez-Sánchez:** Investigation. **Margarita L Martínez-Fierro:** Investigation. **Concepción Cordero-Chaclan:** Methodology. **Yaliana Tafurt-Cardona:** Writing – review & editing, Writing – original draft. **Paúl Mendoza Pérez:** Writing – review & editing, Writing – original draft. **Alejandra de Jesús Joo-Domínguez:** Project administration. **Rodrigo De la Cruz-Calderón:** Investigation. **Marisol Espinoza-Ruiz:** Investigation. **Consuelo Chang-Rueda:** Investigation. **Fanny Carmina Lee-Faviel:** Investigation.

## Funding

This research was funded by the Consejo Nacional de Humanidades, Ciencia y Tecnología (CONAHCYT, Mexican National Council for Humanities, Science and Technology), grant number Salud-2012-01-180845.

## Declaration of Competing Interest

The authors declare the following financial interests/personal relationships which may be considered as potential competing interests: Karina del Carmen Trujillo Murillo reports financial support was provided by Consejo Nacional de Humanidades, Ciencias y Tecnologías. If there are other authors, they declare that they have no known competing financial interests or personal relationships that could have appeared to influence the work reported in this paper.

## Data Availability

All data supporting the findings of this study are available within the manuscript and its Supplementary Information. Any additional datasets generated or analyzed during the current study are available from the corresponding author upon reasonable request.
