# Supplementary material for: Development and validation of a quantitative pcr array assay for the detection of viral sequences in breast cancer
Source: J Genet Eng Biotechnol. 2026 Apr 16;24(2):100693. doi: 10.1016/j.jgeb.2026.100693 (PMC13099489; doi:10.1016/j.jgeb.2026.100693)
Supplement: Supplementary Data 2 [file mmc2.docx]

**Table 1**. Copy number in each of the prepared dilutions of gBlocks®.

|  |  |  | Dilutions | | | | | | | |
| --- | --- | --- | --- | --- | --- | --- | --- | --- | --- | --- |
| gBlocks® | | | **1** | **2** | **3** | **4** | **5** | **6** | **7** | **8** |
| Length in bp | **Name** | **Copy number per 10 ng** | **1:10e3** | **1:10e4** | **1:10e5** | **1:10e6** | **1:10e7** | **1:10e8** | **1:10e9** | **1:10e10** |
| 180 | HPV18-E1-REF | 5.15E+10 | 5.15E+07 | 5.15E+06 | 5.15E+05 | 5.15E+04 | 5.15E+03 | 5.15E+02 | 5.15E+01 | 5.15E+00 |
| 143 | HPV45-E1-REF | 6.48E+10 | 6.48E+07 | 6.48E+06 | 6.48E+05 | 6.48E+04 | 6.48E+03 | 6.48E+02 | 6.48E+01 | 6.48E+00 |
| 135 | HPV59-E1-REF | 6.86E+10 | 6.86E+07 | 6.86E+06 | 6.86E+05 | 6.86E+04 | 6.86E+03 | 6.86E+02 | 6.86E+01 | 6.86E+00 |
| 135 | HPV68-E1-REF | 6.86E+10 | 6.86E+07 | 6.86E+06 | 6.86E+05 | 6.86E+04 | 6.86E+03 | 6.86E+02 | 6.86E+01 | 6.86E+00 |
| 135 | HPV39-E1-REF | 6.86E+10 | 6.86E+07 | 6.86E+06 | 6.86E+05 | 6.86E+04 | 6.86E+03 | 6.86E+02 | 6.86E+01 | 6.86E+00 |
| 187 | HPV35-E1-REF | 4.95E+10 | 4.95E+07 | 4.95E+06 | 4.95E+05 | 4.95E+04 | 4.95E+03 | 4.95E+02 | 4.95E+01 | 4.95E+00 |
| 160 | HPV31-E1-REF | 5.79E+10 | 5.79E+07 | 5.79E+06 | 5.79E+05 | 5.79E+04 | 5.79E+03 | 5.79E+02 | 5.79E+01 | 5.79E+00 |
| 153 | HPV52-E1-REF | 6.06E+10 | 6.06E+07 | 6.06E+06 | 6.06E+05 | 6.06E+04 | 6.06E+03 | 6.06E+02 | 6.06E+01 | 6.06E+00 |
| 200 | HPV33-E1-REF | 4.63E+10 | 4.63E+07 | 4.63E+06 | 4.63E+05 | 4.63E+04 | 4.63E+03 | 4.63E+02 | 4.63E+01 | 4.63E+00 |
| 150 | HPV58-E1-REF | 6.18E+10 | 6.18E+07 | 6.18E+06 | 6.18E+05 | 6.18E+04 | 6.18E+03 | 6.18E+02 | 6.18E+01 | 6.18E+00 |
| 214 | HPV16-E1-REF | 4.33E+10 | 4.33E+07 | 4.33E+06 | 4.33E+05 | 4.33E+04 | 4.33E+03 | 4.33E+02 | 4.33E+01 | 4.33E+00 |
| 169 | HPV56-E1-REF | 5.48E+10 | 5.48E+07 | 5.48E+06 | 5.48E+05 | 5.48E+04 | 5.48E+03 | 5.48E+02 | 5.48E+01 | 5.48E+00 |
| 150 | EBV-BNRF1-REF | 6.18E+10 | 6.18E+07 | 6.18E+06 | 6.18E+05 | 6.18E+04 | 6.18E+03 | 6.18E+02 | 6.18E+01 | 6.18E+00 |
| 197 | MMTV-REF | 4.70E+10 | 4.70E+07 | 4.70E+06 | 4.70E+05 | 4.70E+04 | 4.70E+03 | 4.70E+02 | 4.70E+01 | 4.70E+00 |
| 200 | CMV-UL48-REF | 4.63E+10 | 4.63E+07 | 4.63E+06 | 4.63E+05 | 4.63E+04 | 4.63E+03 | 4.63E+02 | 4.63E+01 | 4.63E+00 |
| 270 | HTLV1-TAX-REF | 3.43E+10 | 3.43E+07 | 3.43E+06 | 3.43E+05 | 3.43E+04 | 3.43E+03 | 3.43E+02 | 3.43E+01 | 3.43E+00 |
| 270 | HTLV2-TAX-REF | 3.43E+10 | 3.43E+07 | 3.43E+06 | 3.43E+05 | 3.43E+04 | 3.43E+03 | 3.43E+02 | 3.43E+01 | 3.43E+00 |
| 270 | HHV8-UL27-REF | 3.43E+10 | 3.43E+07 | 3.43E+06 | 3.43E+05 | 3.43E+04 | 3.43E+03 | 3.43E+02 | 3.43E+01 | 3.43E+00 |
| 250 | HSV1-UL27-REF | 3.71E+10 | 3.71E+07 | 3.71E+06 | 3.71E+05 | 3.71E+04 | 3.71E+03 | 3.71E+02 | 3.71E+01 | 3.71E+00 |
| 123 | HSV2-UL27-REF | 7.53E+10 | 7.53E+07 | 7.53E+06 | 7.53E+05 | 7.53E+04 | 7.53E+03 | 7.53E+02 | 7.53E+01 | 7.53E+00 |
| 126 | CI+-AatAEcoli | 7.35E+10 | 7.35E+07 | 7.35E+06 | 7.35E+05 | 7.35E+04 | 7.35E+03 | 7.35E+02 | 7.35E+01 | 7.35E+00 |
| 270 | CTRL-HHB | 3.43E+10 | 3.43E+07 | 3.43E+06 | 3.43E+05 | 3.43E+04 | 3.43E+03 | 3.43E+02 | 3.43E+01 | 3.43E+00 |

| 20X PRIMER+PROBE MIX | |
| --- | --- |
| REAGENTS | **Volume** |
| Primer Forward: 100 µM | 9 µL |
| Primer Reverse: 100µM | 9 µL |
| TaqMan VEB-FAM Probe: 100 µM | 2.5 µL |
| Molecular Biology Grade H_2_O | 29.5 µL |
| Final volume | **50 µL** |

**Table 2**. Conditions of the amplification reaction.

| REAGENTS | VOLUME PER REACTION |
| --- | --- |
| Master Mix 2X | 9 µL |
| 20X PRIMER+PROBE MIX | 0.5 µL |
| Molecular Biology Grade H2O | 4.5 µL |
| Final volume | ***10 µL** |
|  | *To this final volume,  1 µL of each dilution was added. |

**Table 3.** Conditions of the amplification program.

| TEMPERATURE | TIME |  |
| --- | --- | --- |
| 95 °C | 3 min |  |
| 95 °C | 3 seg | **40 cycles** |
| 52 °C | 10 seg |  |
| 60 °C | 30 seg |  |
